# Cell-Free DNA Promotes Thrombin Autolysis and Generation of Thrombin-Derived C-Terminal Fragments

**DOI:** 10.3389/fimmu.2021.593020

**Published:** 2021-02-24

**Authors:** Rathi Saravanan, Yeu Khai Choong, Chun Hwee Lim, Li Ming Lim, Jitka Petrlova, Artur Schmidtchen

**Affiliations:** ^1^ Lee Kong Chian School of Medicine, Nanyang Technological University Singapore, Singapore, Singapore; ^2^ Interdisciplinary Graduate School, NTU Institute for Health Technologies, Nanyang Technological University Singapore, Singapore, Singapore; ^3^ Division of Dermatology and Venereology, Department of Clinical Sciences, Lund University, Lund, Sweden; ^4^ Wound Healing Centre, Bispebjerg Hospital, Department of Biomedical Sciences, University of Copenhagen, Copenhagen, Denmark

**Keywords:** thrombin, host defense peptides, cell-free DNA (cfDNA), coagulation, molecular innate immunity, NETs (neutrophil extracellular traps), antimicrobial peptides

## Abstract

Cell-free DNA (cfDNA) is the major structural component of neutrophil extracellular traps (NETs), an innate immune response to infection. Antimicrobial proteins and peptides bound to cfDNA play a critical role in the bactericidal property of NETs. Recent studies have shown that NETs have procoagulant activity, wherein cfDNA triggers thrombin generation through activation of the intrinsic pathway of coagulation. We have recently shown that thrombin binds to NETs *in vitro* and consequently can alter the proteome of NETs. However, the effect of NETs on thrombin is still unknown. In this study, we report that DNA binding leads to thrombin autolysis and generation of multiple thrombin-derived C-terminal peptides (TCPs) *in vitro.* Employing a 25-residue prototypic TCP, GKY25 (GKYGFYTHVFRLKKWIQKVIDQFGE), we show that TCPs bind NETs, thus conferring mutual protection against nuclease and protease degradation. Together, our results demonstrate the complex interplay between coagulation, NET formation, and thrombin cleavage and identify a previously undisclosed mechanism for formation of TCPs.

## Introduction

Neutrophil extracellular traps (NETs) are web-like structures exuded by activated neutrophils as first line of host defense, in response to pathogens and other stimuli (1). Apart from microbes and microbial products, substances such as cytokines, immune complexes, autoantibodies, chemical derivatives all trigger NETs formation or NETosis (2). NET fibers comprise majorly of cell-free DNA (cfDNA) as the structural scaffold, decorated with histones, granule derived enzymes (such as neutrophil elastase and cathepsin G) and antimicrobial proteins and peptides (lactoferrin, lysozyme, α-defensins and LL-37). Such a complex structure with a high local concentration of antimicrobial enables NETs to effectively trap pathogens, contain microbial spread and kill microbial invaders, thus playing a critical role in innate immune defense (1).

Contrasting to the host defense function, the role of NETs in the pathogenesis of inflammatory disorders, autoimmune diseases and intravascular damage is becoming increasingly evident (3). Several studies have reported on the procoagulant activity of NETs. cfDNA triggers thrombin generation *via* activation of the contact pathway and elevated levels of circulating cfDNA is a hallmark feature in patients with deep vein thrombosis and sepsis (4–7).

While the mechanism of cfDNA induced thrombin generation has been the focus of most studies, as such, the interaction of thrombin with NETs remained unclear. In a recent study, we have reported that thrombin binds to NETs possibly through DNA binding *in vitro* and alters the proteome of NETs which may consequently influence its physiological role during infection and immune response (8). Nonetheless, the effect of DNA/NETs on thrombin was not investigated.

To this end, in this study, we investigated the fate of DNA bound thrombin. We show that DNA binding induces thrombin autolysis and release of thrombin-derived C-terminal peptides (TCP), previously shown to have antibacterial activity and immunomodulatory functions *in vitro* and *in vivo* (9–14). Moreover, we demonstrate that a 25-residue prototypic TCP, GKY25, binds to DNA and NETs, which results in increased protease and nuclease stability of peptide and DNA, respectively.

## Materials and Methods

### Ethics Statement

Human whole blood was collected from healthy subjects with written informed consent. Methods involving the use of human whole blood and derived cells were in accordance with the guidelines and regulations approved by the Nanyang Technological University Institutional Review Board, Singapore (IRB-2014-10-041).

### Peptides

The thrombin-derived peptide GKY25 (GKYGFYTHVFRLKKWIQKVIDQFGE), the control peptide IVE25 (IVEGSDAEIGMSPWQVMLFRKSPQE), and LL-37 (LLGDFFRKSKEKIGKEFKRIVQRIKDFLRNLVPRTES) were synthesized by Biopeptide (San Diego, CA). The purity (>95%) of these peptides was confirmed by mass spectral analysis (MALDI-TOF Voyager).

### DNA Isolation

The DNA used for agarose gel and western blot analysis was extracted from cultured human keratinocytes (HaCaT) cells using E.Z.N.A. DNA/RNA Isolation kit (Omega Bio-Tek USA) or *E. coli* DH5α plasmid DNA (pUC19) using Qiagen plasmid purification kits (Qiagen USA) according to the manufacturer’s protocol.

### DNA Gel Shift Assay

20 µg/ml (200 ng) of DNA was incubated with increasing concentrations (10–200 ng) of proteins (thrombin, prothrombin) or peptides (GKY25, IVE25, or LL-37) in 10 mM Tris, pH 7.4 for 1 hour at 37 °C. Following this, the samples were analyzed by gel electrophoresis on a 0.8% agarose (w/v) gel in 1X Tris acetate-EDTA (TAE) buffer with SYBR Safe and visualized with the ChemiDoc Imaging System (Bio-Rad, USA).

### Analysis of Thrombin Generation by Western Blot

10 µl of citrate plasma was added to increasing concentration of DNA (6.25, 12.5, 25, and 50 µg/ml) resuspended in 10 mM Tris, pH 7.4 (total volume of 20 µl). Following an hour pre-incubation at 37 °C, 2.5 mM CaCl_2_ was added to samples and further incubated for 1 hour at 37 °C. After incubation, samples were boiled for 15 min with sample buffer and were analyzed by SDS-PAGE using Novex 10–20% Tris Tricine precast gels. Post electrophoresis, the gel was transferred to a PVDF membrane and blocked with 5% milk for an hour before western blotting. For detection of C-terminal thrombin fragments, overnight incubation of the blots was performed with polyclonal antibodies recognizing the thrombin C-terminal peptide VFR17 (VFRLKKWIQKVIDQFGE) in 1:5,000 dilution at 4 °C. Post washing thrice (3 x 10 mins) with PBS-T the blot was incubated with HRP-conjugated secondary antibodies (Dako) at 1:10,000 dilutions in 5% milk for an hour. After repeating the wash thrice, the membranes were developed using the SuperSignal West Dura Extended Substrate (ThermoFisher Scientific, USA) for 5 min and thereafter imaged using a gel documentation system (Gel Doc XR + System).

### Fluorogenic Assay of Thrombin Generation

Thrombin generation assay was performed using Technothrombin TGA (Technoclone, Vienna, Austria) based on the manufacturing protocol. Briefly, 40 µl citrated human plasma was spiked with increasing concentrations of DNA (0, 5, 10, and 15 µg/ml), and incubated for 2 h at 37 °C. After incubation, 60 µl of reagent (RC Low), 7.5 mM of CaCl_2_ and 0.5 mM of the fluorogenic substrate (Z-Gly-Gly-Arg-AMC) mixture were added and thrombin generation was continuously monitored for 60 min with 1-minute interval at 360 _Ex_/460_Em_ nm. The assay was performed in technical duplicates and thrombin generation profiles were evaluated using the Technothrombin TGA software.

### Thrombin Autolysis Determination

DNA dependent thrombin autolysis was analyzed by incubating 0.4 µg of human thrombin (Innovative Research USA) in the presence or absence of equal weight ratio (1:1) of DNA in a total volume of 10 µl for different time points (1, 2, 4, and 6 h). Likewise, for dose dependent analysis, 0.4 µg of thrombin was incubated for 4 h with increasing concentration of DNA in terms of weight ratio (1:1, 1:5, 1:10). Thrombin autolysis inhibition was analyzed using 0.4 µg of heat inactivated thrombin (preboiled for 10 mins at 95 °C) incubated with equal weight ratio (1:1) of DNA for extended time of 6 h. In another set of incubation, 0.4 µg of thrombin was incubated with equal weight ratio (1:1) of DNA for 4 h, in the presence of PMSF or Protease inhibitor cocktail at a final concentration of 1 mM and 1X respectively. In all cases, after the incubation at 37 °C, samples were boiled for 10 min in sample buffer and analyzed by western blotting as mentioned above.

### Circular Dichroism Spectroscopy

The secondary structure GKY25, in solution and DNA bound form was analyzed by circular dichroism spectra measured using Chirascan Circular Dichroism spectrometer (Applied Photophysics, U.K) using a 1 mm path length cuvette (Hellma). Spectral data were collected with step size of 0.5 and time constant of 1s. The peptide concentration for the measurements was fixed as 20 μM. The peptide was titrated with increasing concentration of DNA in TB and the spectrum was recorded. All spectra were recorded at 25°C from 190 to 260 nm using a bandwidth of 1-nm and averaged over three scans. Baseline scans were obtained using the same parameters for buffer containing respective DNAs and subtracted from the respective data scans with peptides. The final corrected averaged spectra were expressed in mean residue ellipticity.

### Intrinsic Tryptophan Fluorescence

Fluorescence experiments were carried out using Cary Eclipse fluorescence spectrophotometer (Varian, Inc., Australia) equipped with dual monochromators. All measurements were performed using 0.1 cm path length cuvette and a slit width of 5 nm. Fluorescence spectra were recorded in TB. Intrinsic tryptophan fluorescence spectrum of free peptide was recorded using 5 μM peptide. Interaction between peptide and DNA was studied by titrating peptide with equal amount of genomic DNA (gDNA). The intrinsic tryptophan was excited at wavelength of 280 nm and the emission was monitored between 300–400 nm.

### Neutrophil Isolation

Neutrophils were isolated from human whole blood anti-coagulated with sodium citrate. Whole blood was layered on Polymorphprep and centrifuged at 500x *g*, 30 min at room temperature (without brakes). The isolated neutrophil containing plasma sample was then supplemented with Erythrocyte Lysis Buffer (eBioscience, USA) to remove any contaminating erythrocytes. Then, isolated neutrophils were resuspended in RPMI medium supplemented with 10% fetal bovine serum (FBS). To verify the purity of the isolation, neutrophils were stained with Brilliant Violet 510 (BV510)-anti-CD45 (clone HI30), fluorescein isothiocyanate (FITC)-anti-CD66b (clone G10F5) and 4’6-diamidino-2-phenylindole (DAPI) in PBS supplemented with 5% FBS then assessed by flow cytometry with the LSR-Fortessa-X20 (Becton-Dickinson, USA). Live neutrophils were determined as DAPI-negative (>99%) and CD45+CD66b+ (>95%).

### Visualization of GKY25-Neutrophil Extracellular Trap Interaction

Neutrophils (200,000 cells, 5 X 10^5^ cells/ml) were incubated with 25 nM phorbol 12-myristate 13-acetate (PMA; Thermo-Fisher Scientific, USA), 5 µM calcium ionophore A23187 (Sigma Aldrich, USA) or the bacteria *Pseudomonas aeruginosa* at a multiplicity of infection (MOI) of 10 on poly-L-lysine coated coverslips in 24-well plate for 3 h at 37 °C to stimulate NET formation. Then, the supernatants were removed, and the wells were gently washed once with pre-warmed RPMI with 10% FBS. Afterward, 5 µM of TAMRA-labeled IVE25 or GKY25 was added onto the coverslips for a further 1-hour incubation at 37°C. The wells were then gently washed with pre-warmed TBS, fixed with 4% paraformaldehyde and washed again with 0.1% Tween-20 in TBS (TBS-T). The coverslips were finally mounted on DAPI-containing mounting medium Fluoroshield (Thermo-Fisher Scientific, USA) and imaged using the LSM 800 with Airyscan confocal Microscope (Zeiss, Germany).

### Serum Protection Assay

DNA (0.5 µg) was complexed with peptide (5 µg) at room temperature for 1 hour in 10 mM Tris, pH 7.4 buffer (volume 10 µl) containing equal volume of FBS (final concentration 50%). The samples were incubated for 4 h at 37 °C. Equal volume (20 µl) of phenol chloroform was added to the samples and spun at high speed for 5 min. The separated top aqueous phase was transferred to a microfuge tube, followed by adding 3 volumes of ice-cold ethanol, and placed in -80 °C for at least 1 hour. The tube was centrifuged at high speed for 30 min, washed with 70% ethanol for 3 times, and resuspended in buffer or water. Samples were loaded to 0.4% gel stained with SYBR safe and run at 90V for about 30 min.

### Protease Protection Assay

Thrombin or GKY25 (0.4 µg) was incubated with DNA (w/w ratio = 1:1) in 10 mM Tris, pH 7.4 for 4 h, followed by addition of neutrophil elastase at enzyme: substrate ratio of 1:30 and incubated for various time points (1, 2, and 3 h). Post incubation, samples were boiled for 3 min to inhibit the reaction and further analyzed by Tricine SDS-PAGE as described above.

### DNase I Protection Assay

DNA (0.5 µg) was complexed with GKY25 (5 µg) at RT for 1 hour in 10 mM Tris, pH 7.4 in a total volume of 10 µl. DNase I was added to a final concentration of 0.1U and incubated for 1 hour at 37 °C. Post incubation, the samples were boiled at 75 °C for 10 min to stop the reaction, followed by addition of Proteinase K (1 µg) for digestion of the peptide. Samples were incubated at 55 °C for 90 min and analyzed by agarose gel (0.4%) and run at 90V for about 30 min and stained with SYBR safe.

### Fluorescence-Based DNA Quantification

The fluorescent DNA-chelating dye Sytox Green (Thermo-Fisher Scientific, USA) was used to stain free DNA for quantification. DNA (approx. 2.47 µg/ml) was incubated with the indicated concentrations of GKY25 for 30 min at 37°C. Then, either buffer or 0.2 U/ml MNase was added to the mixture for 15 min at 37°C (DNA concentration = ~2.22 µg/ml). The DNA-GKY25-MNase mixture was then heat-inactivated for 30 min at 65°C and iced immediately thereafter. Finally, 0.25 mg/ml proteinase K was added for another incubation for 90 min at 37°C (DNA concentration = ~ 2.00 µg/ml). The complete reaction solution was added 1:1 with 100 nM Sytox Green (duplicate) in a black, flat-bottom 96-well plate and incubated in the dark for 15 min at room temperature (final DNA concentration = ~ 1.00 µg/ml; working Sytox Green concentration = 50 nM). The fluorescence was read at 480_Ex/_530_Em_ using the Cytation 3 Cell-Imaging Multi-mode reader (BioTek, USA).

### Statistical Analysis

All experiments were repeated at least three times unless otherwise stated. Data are presented as means +/− SD. Statistical analysis was performed using GraphPad Prism software v6/v7 (GraphPad, USA). For multiple comparison either one-way ANOVA with Dunnett’s or two-way ANOVA with tukey´s test was used. Statistical significance based on p values were *p < 0.05, **p < 0.01, *** p < 0.001.

## Results

### Cell-Free DNA Promotes Thrombin Formation and Thrombin Binds DNA

**Figure 1:**
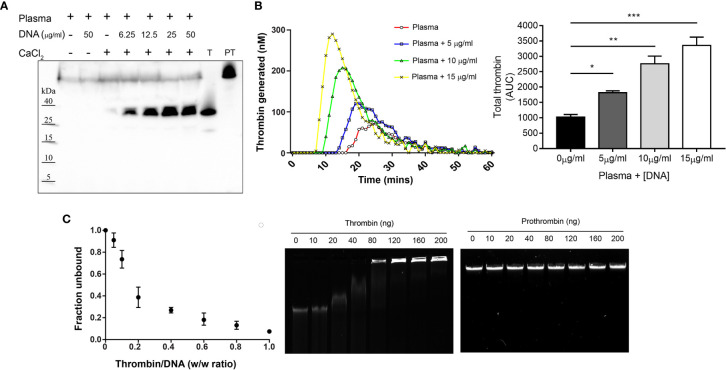
Cell-free DNA promotes thrombin generation and thrombin binds DNA. **(A)** Western blot analysis showing formation of thrombin in human citrate plasma (10 µl) upon addition of increasing concentration of DNA (6.25, 12.5, 25 and 50 µg/ml) resuspended in 10 mM Tris, pH 7.4 containing CaCl_2_ (2.5 mM) to activate coagulation. Prothrombin (PT) and thrombin (T) alone were used as control. Blot shown is representative of at least three independent experiments. **(B)** Fluorogenic thrombin generation assay demonstrating effect of genomic DNA (gDNA) on total thrombin generated (area under the curve – AUC) measured in plasma. Data represents the mean ± SD of samples run in technical duplicates. Statistical analysis performed using one-way ANOVA with Dunnett’s test. *p < 0.05, **p < 0.01, ***p < 0.001. **(C)** Gel retardation analysis of increasing weight ratio (0, 10, 20, 40, 80, 120, 160, 200 ng) of thrombin and prothrombin interaction with gDNA (200 ng). The graph (left) shows the unbound fraction of DNA as a function of thrombin weight ratio. Data represents the mean ± SD of n = 3 independent experiments. Agarose gel shown is representative of three independent experiments.

Based on the earlier studies, we set out to assess the procoagulant effect of cell-free DNA on *in vitro* thrombin generation. Western blot analysis of human plasma incubated with DNA showed increased thrombin formation, compared to plasma only control (**Figure 1A**). Additionally, thrombin generation was monitored using a fluorogenic thrombin generation assay. **Figure 1B** shows changes in the concentration of thrombin formed as a function of time. In the presence of DNA, the lag time and thrombin peak time was shorter, and the concentration of thrombin increased compared to the thrombogram for plasma alone. With increased DNA concentration, a drastic reduction in lag time and peak time as well as a corresponding DNA dose-dependent increase in thrombin concentration was observed (**Figure 1B**).

Following this, we analyzed the DNA binding ability of thrombin using an agarose gel mobility shift assay. Reduced DNA migration was observed with addition of 40 ng of thrombin and complete retardation occurred at or above 160 ng of thrombin. Interestingly, the precursor prothrombin showed no changes in gel retardation, indicating that DNA interaction and binding ability was exclusive to active thrombin (**Figure 1C**). These results indicate that cell-free DNA augments thrombin formation and active thrombin binds to DNA, findings consistent with previous reports (4, 7).

### DNA Binding Triggers Thrombin Autolysis and Thrombin Derivative Formation

**Figure 2:**
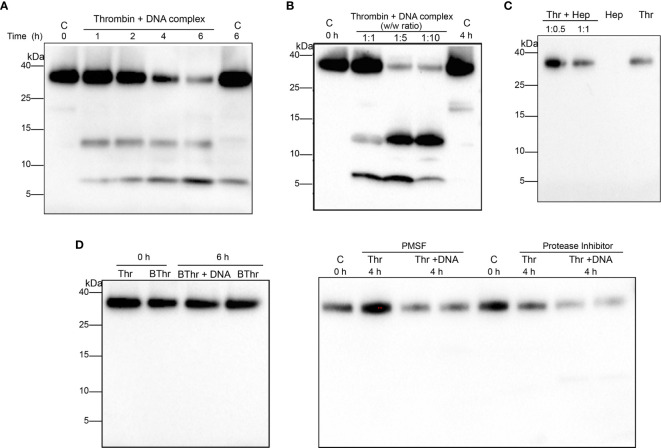
DNA binding stimulates thrombin autolysis and generates thrombin C-terminal peptides. Western blot analysis of human thrombin (0.4 µg) **(A)** incubated with equal weight ratio of DNA for increasing time points **(B)** thrombin incubated for 4 h with increasing weight ratios of DNA. Thrombin alone directly or after incubation at 37 °C was used as a control. **(C)** Thrombin incubated with different weight ratio of heparin (1:0.5, 1:1) for 1 h at 37 °C and analyzed using western blotting. Heparin (Hep) or thrombin (Thr) alone (0.4 µg) was used as control. **(D)** Heat inactivated thrombin (0.4 µg) or thrombin incubated with equal weight ratio of DNA in the presence of PMSF (1 mM) or protease inhibitor cocktail (1X) at 37 °C and analyzed using western blotting. Heat inactivated thrombin or thrombin alone was used as control. All data shown is representative of at least five independent experiments.

Next, we investigated the effect of DNA binding on thrombin. Western blot analysis of thrombin complexed with DNA showed the formation of two prominent TCP fragments having molecular masses of ~ 12 kDa and ~7 kDa (**Figure 2A**). Prolonged incubation showed reduced intensity of thrombin and a corresponding increased intensity of the TCP fragments, suggesting that DNA induced thrombin fragmentation. To validate this further, thrombin was incubated with increasing concentrations of genomic DNA (**Figure 2B**) and TCP generation was also observed. Interestingly, incubation with intact NETs resulted in a similar TCP pattern, however, the intensity of the formed TCP fragments was lower (**Supplementary Figure 1A**).

Moreover, PMSF (a serine protease inhibitor) or PI (a cocktail of protease inhibitors) as well as addition of heat denatured thrombin abrogated the formation of TCPs (**Figure 2D**), excluding the possibility that other unknown contaminating proteases were responsible for generation of the observed TCPs, confirming that the fragmentation was primarily due to induction of thrombin autoproteolysis. Furthermore, to investigate whether the observed thrombin fragmentation and TCP formation was induced by other polyanionic polymers of higher charge, thrombin was incubated with heparin, a known ligand of the thrombin anion binding exosite II (ABE II). The results showed that heparin did not induce thrombin fragmentation or TCP formation at the dose studied (**Figure 2C**). Taken together our results demonstrate that binding to DNA component of NETs triggers thrombin autolysis, yielding thrombin derivatives and TCP fragments.

### Thrombin C-Terminal Peptides Bind Neutrophil Extracellular Traps

**Figure 3:**
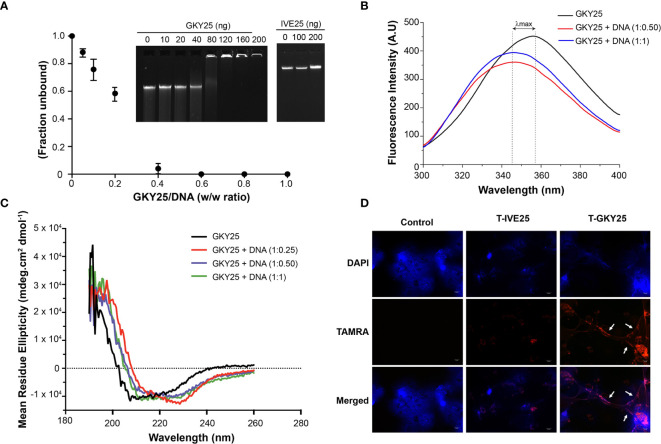
Thrombin C-terminal peptides bind DNA. **(A)** Gel retardation analysis of increasing weight ratio (0, 10, 20, 40, 80, 120, 160, 200 ng) of GKY25 (left) and IVE25 (right) with gDNA (200 ng). The graph shows the unbound fraction of DNA as a function of GKY25 weight ratio. Data represents the mean ± SD of three independent experiments. Agarose gel shown is representative of three independent experiments. **(B)** Intrinsic fluorescence spectrum of GKY25 in the absence and presence of gDNA **(C)** Circular dichroism analysis of GKY25 alone or in complex with different weight ratios of gDNA. **(D)** Representative fluorescent micrographs of polymorphonucleated cells were treated with PMA only or along with TAMRA labelled GKY25, IVE25 for 3 h at 37 °C. Scale bar = 10 µm.

Considering that thrombin binds DNA and thrombin C-terminal peptides (TCPs) are generated following autolysis, we examined possible TCP-DNA interactions and the resulting structural changes. To this end, a prototypic TCP, GKY25 (12) was incubated with purified genomic DNA and analyzed by a gel mobility shift assay. The results showed reduced DNA migration with addition of 80 ng of the peptide and complete retardation at or above 120 ng of GKY25. In contrast, the control peptide IVE25, derived from the N-terminus of thrombin did not retard DNA (**Figure 3A**). The GKY25-DNA interaction was further analyzed using biophysical methods (**Figures 3B, C**). Intrinsic tryptophan fluorescence emission spectrum of GKY25 showed large blue shifts and associated fluorescence quenching upon titration with DNA (**Figure 3B**), indicating peptide binding to DNA.

Furthermore, circular dichroism spectra showed that DNA binding induces a structural change and the peptide backbone adopts a helical confirmation (**Figure 3C**). In addition, using TAMRA-labelled peptides, we observed that GKY25, not IVE25, co-localized with the extracellular NET structures (**Figure 3D**). Moreover, to study the influence of other stimuli (15, 16) on the binding of GKY25 to NETs, we repeated the immunofluorescence assay using the calcium ionophore A23187 and *P. aeruginosa* as NET inducers. The results (**Supplementary Figure 2**) shows that TAMRA-labelled GKY25 co-localizes to NETs induced by these two stimuli, similar to the observed PMA induced NET binding by GKY25. Together, these data clearly demonstrate that C-terminal fragments derived from the cationic heparin binding exosite II region of thrombin have the ability to bind to the DNA scaffold of NETs.

### DNA Binding Protects Thrombin-Derived C-Terminal Peptides From Protease Degradation

**Figure 4:**
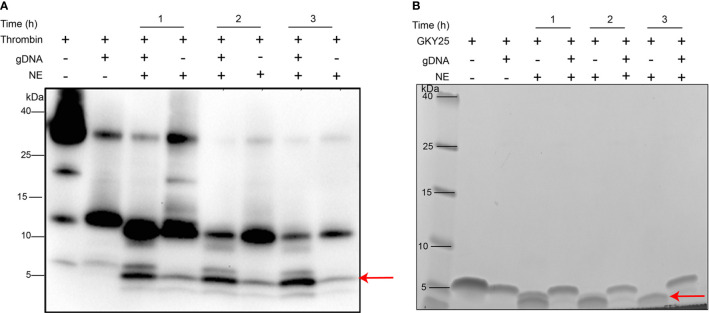
DNA binding protects thrombin C-terminal peptides against protease degradation. **(A)** Western blot analysis of human thrombin (0.4 µg) or **(B)** SDS-PAGE analysis of GKY25 (0.4 µg) complexed with equal weight ratio of gDNA (1:1) and incubated with human neutrophil elastase at various time points (60, 120, and 180 mins) at 37°C. Thrombin alone or GKY25 alone was used as control. The arrow indicates peptides of similar molecular mass (~3 kDa) as the endogenous HVF18 being protected in the presence of DNA. Data shown is representative of three independent experiments.

Neutrophil elastase (NE) has been frequently identified as a NET-associated protein and shown to be biologically active (8, 17). Moreover, as previously mentioned in the Introduction, proteolysis of thrombin by NE releases TCPs with immunomodulatory and anti-microbial activity (9–12). Based on these findings, we therefore analyzed the susceptibility of the generated TCPs to human neutrophil elastase in the presence and absence of DNA (**Figure 4**). As anticipated, in control lanes (without DNA) NE cleaved thrombin to release multiple TCPs, such as low molecular weight TCPs (~3-kDa) similar to HVF18 and FYT21, previously reported to block pro-inflammatory cytokine responses *via* neutralization of LPS and interference with CD14 signaling (9, 10, 18). Interestingly, with increasing incubation time, a higher signal intensity of the 3 kDa TCP was particularly observed in lanes containing DNA (**Figure 4A**), suggesting that the longer TCPs are likely susceptible to NE, generating shorter TCP fragments which are bound to DNA and thereby protected from proteolysis. To explore this further, DNA complexed with GKY25 was incubated with NE at various time points. As shown in **Figure 4B**, a peptide corresponding to intact GKY25 was seen in the presence of DNA, while in the absence of DNA a degraded, faster migrating band was observed. Together, the results indicate that DNA binding protects TCPs from protease degradation and the extent of protection is higher for smaller TCP fragments than larger TCPs.

### Thrombin-Derived C-Terminal Peptide Binding Protects DNA From Nuclease Degradation

**Figure 5:**
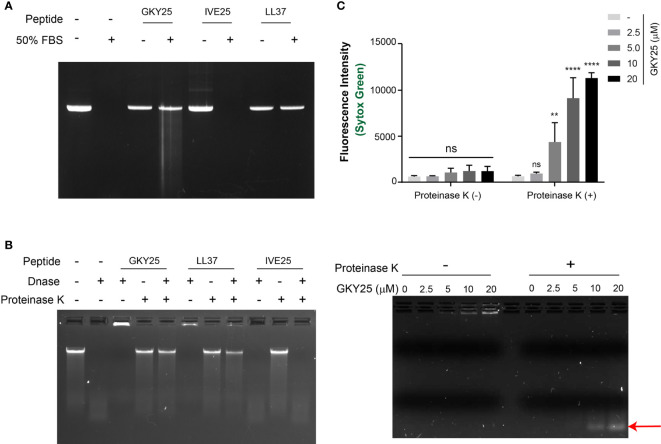
Thrombin C-terminal peptide protects DNA against degradation by nucleases. **(A)** Serum stability assay of gDNA complexed with peptides (GKY25, IVE25 and LL37) incubated in 50% FBS for 4 h at 37°C. After incubation, the DNA was recovered through phenol chloroform extraction and ice-cold ethanol wash as described in materials and methods and analyzed by agarose gel analysis. **(B)** Nuclease protection assay of DNA-peptide complexes incubated with DNAse I (0.1 U) for 1 h at 37°C. Post incubation, the samples were incubated at 75 °C for 10 min to stop the reaction, followed by addition of Proteinase K (1 µg) for digestion of the peptide and further analyzed by agarose gel electrophoresis. In both assays, DNA-peptide complexes were in 1:10 weight ratio. DNA alone was used as control. Data shown is representative of three independent experiments. **(C)** Micrococcal nuclease protection assay of DNA (~2 µg/ml) complexed with increasing concentration of GKY25 and digested with micrococcal nuclease (0.2 U/ml) for 15 min at 37 °C. Post heat-inactivation, the samples were further incubated with proteinase K (0.25 mg/ml) for 90 min at 37°C and analyzed by SYTOX green quantification (top) and agarose gel electrophoresis and (bottom). Arrow indicates DNA fragments protected against micrococcal nuclease digestion by GKY25 at high concentrations. Data represents the mean ± SD of three independent experiments. **p < 0.01, ****p < 0.001 by two-way ANOVA with tukey’s test.

Serum contains heat-stable nucleases shown to degrade DNA wherein DNase I is generally regarded as the major serum nuclease (19, 20). Therefore, we investigated the effect of TCP binding to DNA, specifically in terms of protection against nuclease degradation, as assessed by agarose gel analysis (**Figure 5**). As shown in **Figure 5A**, active nucleases in serum degrade DNA completely in the absence of peptide, whereas presence of GKY25 protects DNA from serum nuclease activity. Likewise, the DNAse I protection assay (**Figure 5B**) showed that GKY25, but not IVE25, efficiently protects DNA against nuclease degradation.

To further validate the protective effect of GKY25 on DNA, the fluorescent dye SYTOX Green based assay was utilized to quantify DNA content (**Supplementary Figure 3**). SYTOX Green is a high-affinity, DNA intercalating dye with high fluorescence intensity in DNA bound state compared to the free dye in solution and hence used in protein-DNA interaction studies (21). Initially, a dose-dependent reduction in fluorescence intensity was observed as a function of increased GKY25 concentration (**Supplementary Figure 2B**), corresponding to GKY25-DNA complex formation and retarded gel migration (**Supplementary Figure 2A**), suggesting a possible competitive DNA binding between GKY25 and the SYTOX Green dye. Subsequent addition of proteinase K restored the DNA gel migration pattern and fluorescence signal (**Supplementary Figure 2**), indicating that proteolysis of GKY25 by proteinase K yielded DNA chains now accessible for SYTOX Green binding.

Using this setup, micrococcal nuclease (MNase) was added to GKY25-DNA complexes before the addition of proteinase K (**Figure 5C**). Here, gel electrophoresis shows increasing DNA content stuck in the loading wells with increasing GKY25 concentrations (**Figure 5C**, bottom left). Removal of GKY25 by proteinase K revealed that the ‘protected’ DNA were lower in molecular weight (**Figure 5C**, bottom right). These ‘protected’ DNA were then subjected to SYTOX Green staining which reflected the increasing DNA contents conferred by increasing GKY25 amounts (**Figure 5C** bottom). Together, the results demonstrate that TCPs interact with DNA and protect DNA against nucleases.

## Discussion

The key novel finding of the present study is that binding of thrombin to cell-free DNA triggers its autoproteolysis, generating TCPs. In addition, we demonstrate that TCPs bind NETs and protect DNA from nuclease degradation. We also show that the TCP-DNA interaction protects low molecular weight TCPs of 2–3 kDa against protease degradation. The findings are of relevance in the context of prothrombin activation and thrombin generation in various inflammatory conditions involving neutrophil chemotaxis, activation and NETs release.

Thrombin is the major serine protease of the blood coagulation system that has both procoagulant and anticoagulant functions (22). Thrombin, through well-orchestrated sequential and rapid activation steps, converts soluble fibrinogen to fibrin and by activating factor XIII crosslinks fibrin monomers yielding stabilized fibrin clot (23). Apart from these fundamental roles in hemostasis, thrombin exerts other physiologic functions (24). It mediates clot stabilization by activation of TAFI and activates transglutaminase (FXIII), causing platelet aggregation due to PAR cleavage (22, 25). Moreover, thrombin elicits numerous cellular responses, including increased CAM expression and growth factor and cytokine release by endothelial cells, as well as growth stimulation of both smooth muscle and fibroblast cells (25). Hence, thrombin´s roles expand to physiological situations involving not only hemostasis, but also immunomodulation and tissue regeneration (26). In this perspective, it is interesting to note that TCPs in the size range of 2–11 kDa indeed are present in human wound fluids as well as fibrin sloughs from acute as well as infected wounds (9, 11, 27). Given that wound healing involves early neutrophil migration to the wound areas and activation and NET formation, future studies should be directed at addressing whether TCPs may be generated also *in vivo* by the DNA-induced autolysis mechanism described here.

From a structural perspective, human α-thrombin autolysis yields β- and γ-thrombin forms, where β-form is the intermediate with subsequent hydrolysis yielding γ-thrombin (28). Our results show that binding to DNA/NETs triggers thrombin autolysis yielding two predominant TCPs, a 12-kDa TCP and 7-kDa TCP (**Figure 2**). Considering the molecular mass similarity and the autolytic cleavage pathway, it is likely that the 12-kDa TCP fragment corresponds to the B4 fragment of γ-thrombin with a molecular mass of 11.8 kDa whereas the 7-kDa fragment represents a product of further hydrolysis. Western blot analysis of γ-thrombin complexed to DNA (**Supplementary Figure 1B**) indeed demonstrates that the 7-kDa TCP is a subsequent hydrolyzed product of the longer 12-kDa TCP. Although further studies are required to precisely identify the formed TCPs, the observation that a γ-thrombin like TCP fragment is formed upon binding cfDNA together with studies showing that γ-thrombin has impaired fibrinogen activity (29, 30) suggests the possibility of a switch in thrombin function - from procoagulant to non-coagulant activity upon DNA binding.

Thrombin autolysis is a slow process wherein complete conversion to γ-thrombin requires nearly 144 h and α-thrombin has been shown to undergo autolysis upon long term storage (31). Previous studies have shown that the rate of autolysis increases with concentration of thrombin, pH, temperature and concentration of monovalent cations (31, 32). Interestingly, our results demonstrate that DNA binding augments thrombin autolysis and TCP formation within 60 min of complexation (**Figure 2A**). Furthermore, we also demonstrate that thrombin autolysis increases with DNA concentration (**Figure 2B**), and that cell-free DNA facilitates thrombin generation. Thus, the present work, demonstrating that cfDNA not only facilitates thrombin generation but also triggers autolysis of thrombin and TCP formation, discloses a previously unknown effect of cfDNA on thrombin.

**Figure 6:**
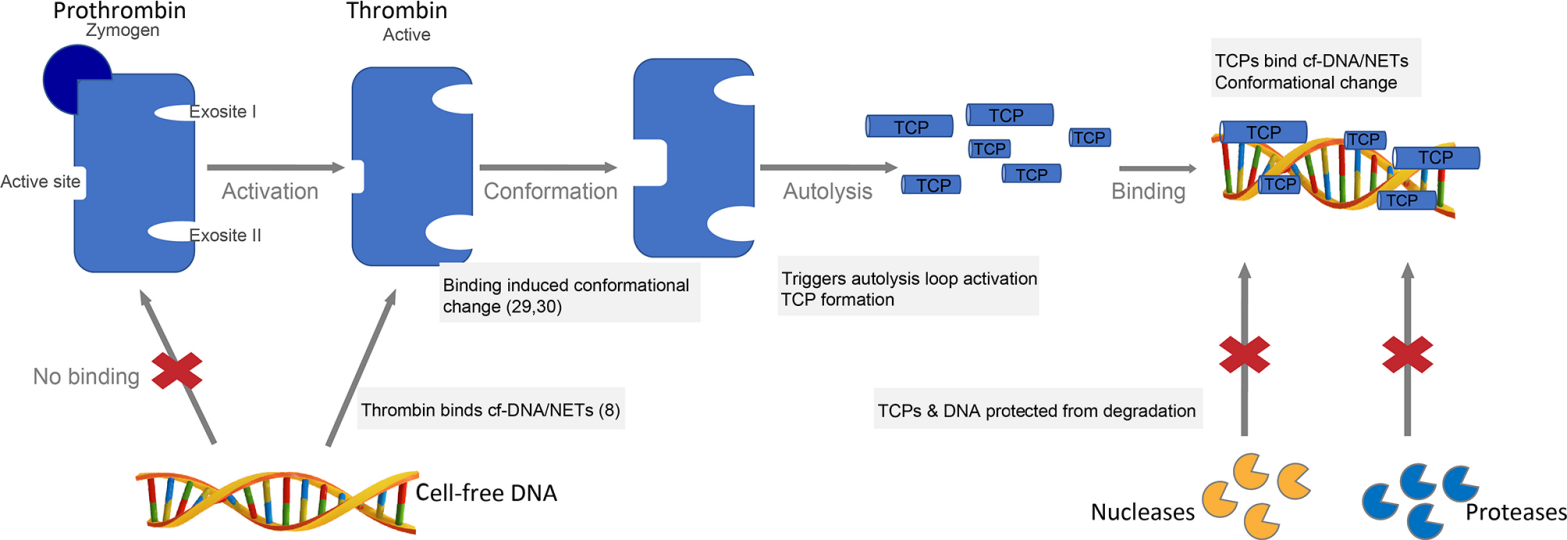
Proposed mechanistic model of DNA binding and DNA-induced thrombin autolysis. Thrombin, the major enzyme of the coagulation cascade is formed by the activation of the zymogen prothrombin (22). Thrombin has two anion binding exosites (ABE) at the opposite sides of the active site and a flexible autolysis loop contributing to substrate specificity (28, 29). Thrombin binds cell-free DNA (cfDNA) and NETs *in vitro* (8), likely *via* the highly cationic exosites. Ligand binding to exosites induces conformational changes (33, 34), which in turn triggers the autolysis loop activation and subsequent TCP formation. TCPs bind cfDNA or NETs yielding protection against degradation by proteases and nucleases, respectively.

Thrombin is a more selective enzyme than most serine proteases owing to the unique positioning of the two anion binding exosites (ABE) at the opposite sides of the active site and a flexible autolysis loop contributing to substrate specificity (28, 29). Given the highly cationic nature of the exosites (fibrinogen binding exosite I and heparin-binding exosite II) and anionic nature of DNA, it is likely that thrombin binds DNA *via* these two exosites. Along this line, our finding that the zymogen prothrombin lacks DNA binding ability (**Figure 1C**) demonstrates that thrombin DNA binding certainly involves the exosites, initially encrypted in the inactive precursor prothrombin and later exposed in active thrombin. Moreover, our observation of DNA induced autolysis and TCP formation in γ-thrombin, known to lack exosite I confirms the involvement of the highly cationic exosite II in thrombin-DNA interactions. Considering that ligand binding to exosites has been shown to influence the conformational properties of thrombin (33, 34), we propose a mechanistic model wherein thrombin binding to DNA *via* the exosites induces conformational changes, thus triggering the autolysis loop activation and subsequent thrombin fragmentation (**Figure 6**). CD results showing peptide backbone conformational changes in the prototypic TCP, GKY25 (encompassing exosite II region) upon binding DNA (**Figure 3**) supports such a possible mechanism. Interestingly, our western blot results with heparin (**Figure 2C**), a well-known ligand of exosite II shows that such a ligand induced autolysis activation could be substrate specific. Of note is the increasing structural evidence demonstrating influence of ligand specific local and long-range communication between thrombin exosites on thrombin function (33). Nonetheless, future studies focusing on delineating DNA binding affinity, binding domain, binding sequence, specificity, binding kinetics as well as autolysis studies in the presence of thrombin inhibitors are essential in understanding the mechanism of DNA induced thrombin autolysis.

Proteolysis of thrombin by neutrophil elastase has been shown to modulate thrombin function (35). Here, we show that the γ-thrombin like TCP fragment generated by autolysis is further proteolyzed by neutrophil elastase to 11-kDa TCP and low molecular weight ~3-kDa TCP (**Figure 4A**), which are present in wound fluids. The aggregation prone 11-kDa TCP forms amyloid-like structures with bacterial LPS and gram-negative bacteria thereby aiding in containment and robust clearance of invading bacteria and bacterial products (11, 13). As previously reported, the smaller TCPs (~3 kDa) exert anti-endotoxic as well as anti-inflammatory effects *in vitro* and *in vivo* by neutralizing LPS and blocking LPS induced pro-inflammatory cytokine response (12, 36, 37). Moreover, TCPs have been shown to interfere with coagulation, controlling contact activation and tissue factor-mediated clotting *in vitro*, leading to normalization of coagulation responses *in vivo* models of sepsis (10). Based on these findings, the formation of such immunomodulatory antimicrobial TCPs suggests that thrombin autolysis and proteolysis jointly modulate thrombin function from coagulation toward host defense and immunomodulation. This is of importance considering the elevated levels of thrombin produced during coagulation and the vital role of thrombin in wound healing (26). On the contrary, overactivation of thrombin autolysis yielding γ-thrombin with impaired fibrinogen activity and platelet aggregation may impair hemostasis and wound healing. Further investigations are warranted to address the effect of DNA-induced thrombin autolysis on the coagulation cascade and other physiologic functions.

Another interesting finding is that TCPs bind to NETs and protect DNA against nuclease degradation (**Figure 5**). The DNA stabilizing effect of TCPs likely correlates to the high cationic nature of host defense peptides (HDPs) as reported earlier for LL-37, human neutrophil peptide-1 (HNP-1) and human beta defensin-3 (hBD-3) (38). It is generally believed that antimicrobial peptides bound to NETs contribute to the direct antimicrobial activity of NETs (1, 3) and hence, although beyond the scope of the current study, further investigations are warranted to evaluate the antimicrobial activity of DNA bound TCPs. Next, considering that serum contains heat-stable nucleases which degrade DNA (19, 20) and invading bacteria secrete nucleases to overcome host defense mechanisms (39, 40), our finding that TCPs stabilize DNA against nuclease degradation suggest that TCPs may contribute to enhanced NETs stability. Furthermore, the co-existence of the aggregation prone 11-kDa TCPs and NETs in the local wound milieu may boost antimicrobial clearance. Thus, TCPs binding to NETs may contribute to the overall enhanced antimicrobial activity of NETs *via* either direct killing, containment, or increased phagocytosis of invading pathogens. In addition, we find that DNA binding offers mutual protection for TCPs against neutrophil elastase (**Figure 4B**) of importance considering the high local concentration of proteases within wound milieu. Moreover, this highlights that NETs could act as natural scaffold for antimicrobial peptides in a proteolytic wound environment. Together with our recent report showing that thrombin alters the proteome of NETs (8), our findings suggest that TCP generation and binding to NETs may affect the overall role of NETs in infection, inflammation, and host response.

In conclusion, our study reports on a previously unknown effect of cell-free DNA on thrombin, wherein cfDNA triggers both thrombin generation as well as thrombin autolysis and generation of TCPs, thus demonstrating a previously undisclosed link between extracellular DNA, thrombin generation and autolysis, and generation of TCPs.

## Data Availability Statement

The raw data supporting the conclusions of this article will be made available by the authors, without undue reservation.

## Ethics Statement

The studies involving human participants were reviewed and approved by Nanyang Technological University Institutional Review Board, Singapore (IRB-2014-10-041). The patients/participants provided their written informed consent to participate in this study.

## Author Contributions

RS, CL, YC, and AS participated in the planning, design, and interpretation of the experiments, results, and validation. CL contributed to the NET preparation, fluorescence microscopy, and nuclease protection assays. JP contributed to the biophysical studies on TCP and DNA interactions. LL and YC performed the Western blots and EMSA experiments. RS and AS wrote the manuscript. All authors reviewed the manuscript. All authors contributed to the article and approved the submitted version.

## Funding

This work was supported by the Lee Kong Chian School of Medicine, Nanyang Technological University Singapore Start-Up Grant, the Singapore Ministry of Education Academic Research Fund Tier 1 (2015-T1-001-82), and the Swedish Research Council (project 2017-02341). RS was supported by the Lee Kong Chian School of Medicine Postdoctoral Fellowship 2014 (L0491020). CL was supported by NTU Institute for Health Technologies, Interdisciplinary Graduate School, Nanyang Technological University Singapore.

## Conflict of Interest

The authors declare that the research was conducted in the absence of any commercial or financial relationships that could be construed as a potential conflict of interest.

The reviewer HK declared a shared affiliation, with no collaboration, with several of the authors, CL, JP, and AS, to the handling editor at the time of review.
