# Supplementary material for: Cell-Free DNA Promotes Thrombin Autolysis and Generation of Thrombin-Derived C-Terminal Fragments
Source: Front Immunol. 2021 Feb 24;12:593020. doi: 10.3389/fimmu.2021.593020 (PMC7943729; doi:10.3389/fimmu.2021.593020)
Supplement: Supplementary file 1 [file DataSheet_1.docx]

**Supplementary Data**

**Cell-Free DNA promotes thrombin autolysis and generation of thrombin-derived C-terminal fragments**

Rathi Saravanan, Yeu Khai Choong, Chun Hwee Lim, Li Ming Lim, Jitka Petrlova,

Artur Schmidtchen

Supplementary Figure 1

^
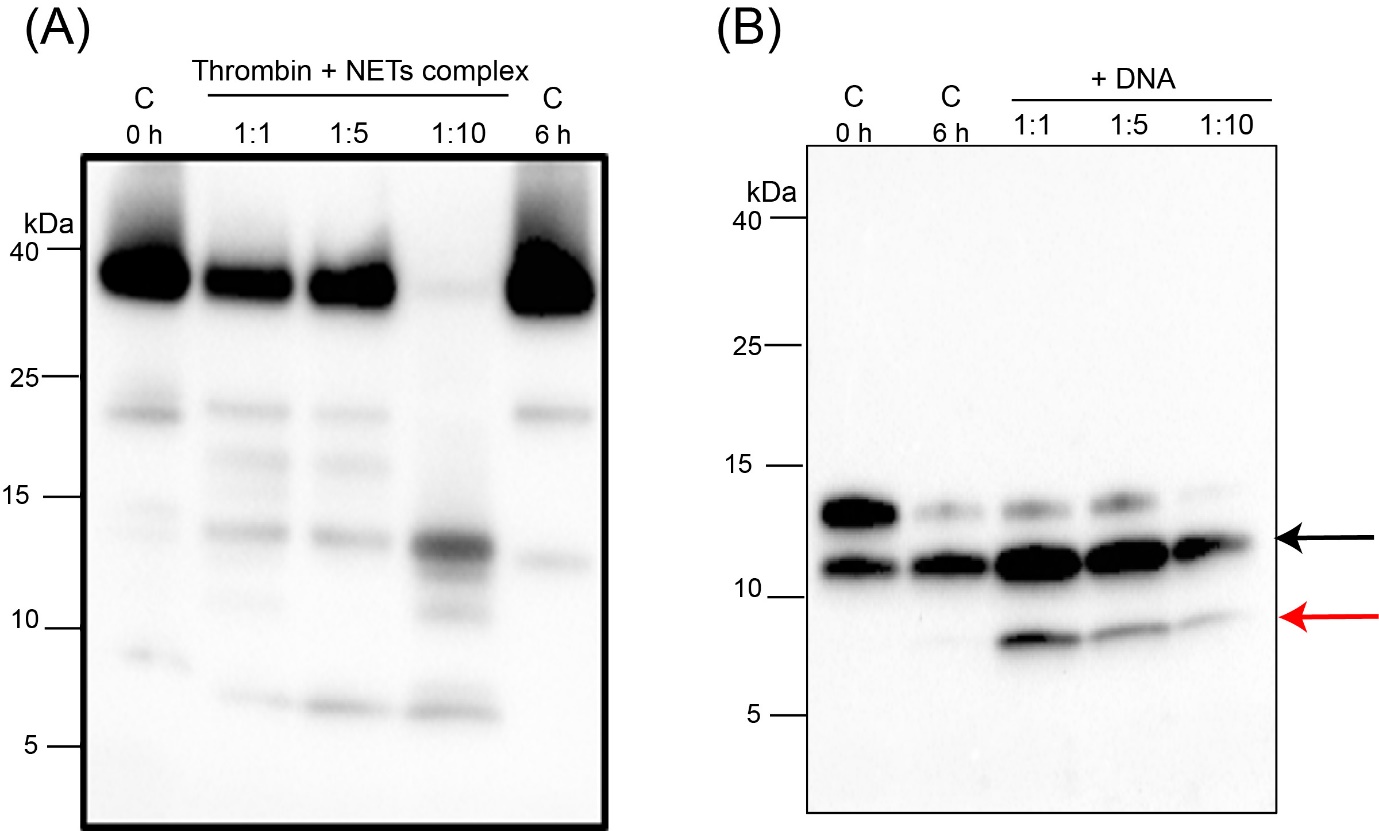
^

**Supp Figure 1. DNA binding stimulates thrombin autolysis yielding thrombin C-terminal peptides.** (A) Western blot analysis of human α-thrombin (0.4 μg) incubated for 4 h at 37 ^o^C with increasing weight ratios of NETs isolated from activated neutrophils. Thrombin alone directly or after incubation at 37 ^o^C was used as control. (B) Western blot analysis of human γ-thrombin (0.4 μg) incubated for 4 h at 37 ^o^C with increasing weight ratios of gDNA. γ-thrombin alone directly or after incubation at 37 ^o^C was used as control. Black arrow corresponds to the B4 fragment of γ-thrombin with a molecular mass of 11.8 kDa and the red arrow corresponds to the hydrolysed TCP fragment (~ 7-kDa).

Supplementary Figure 2


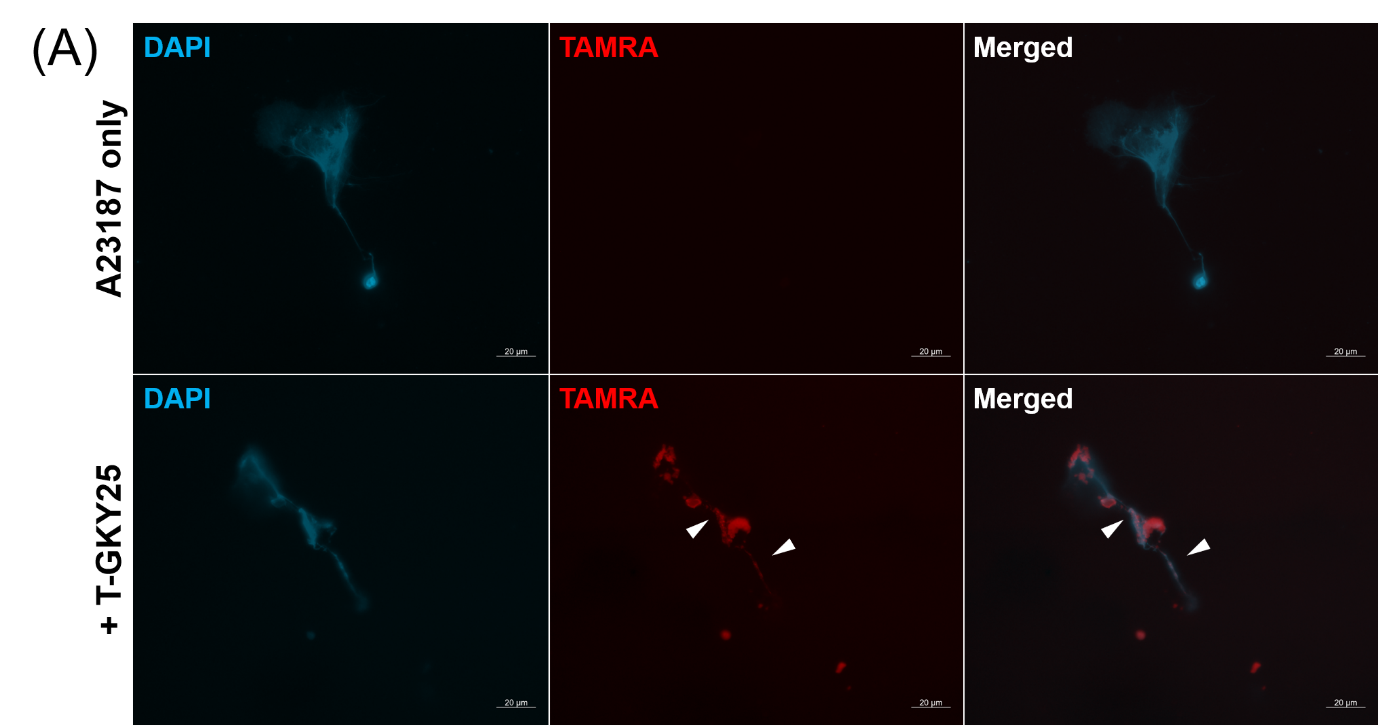

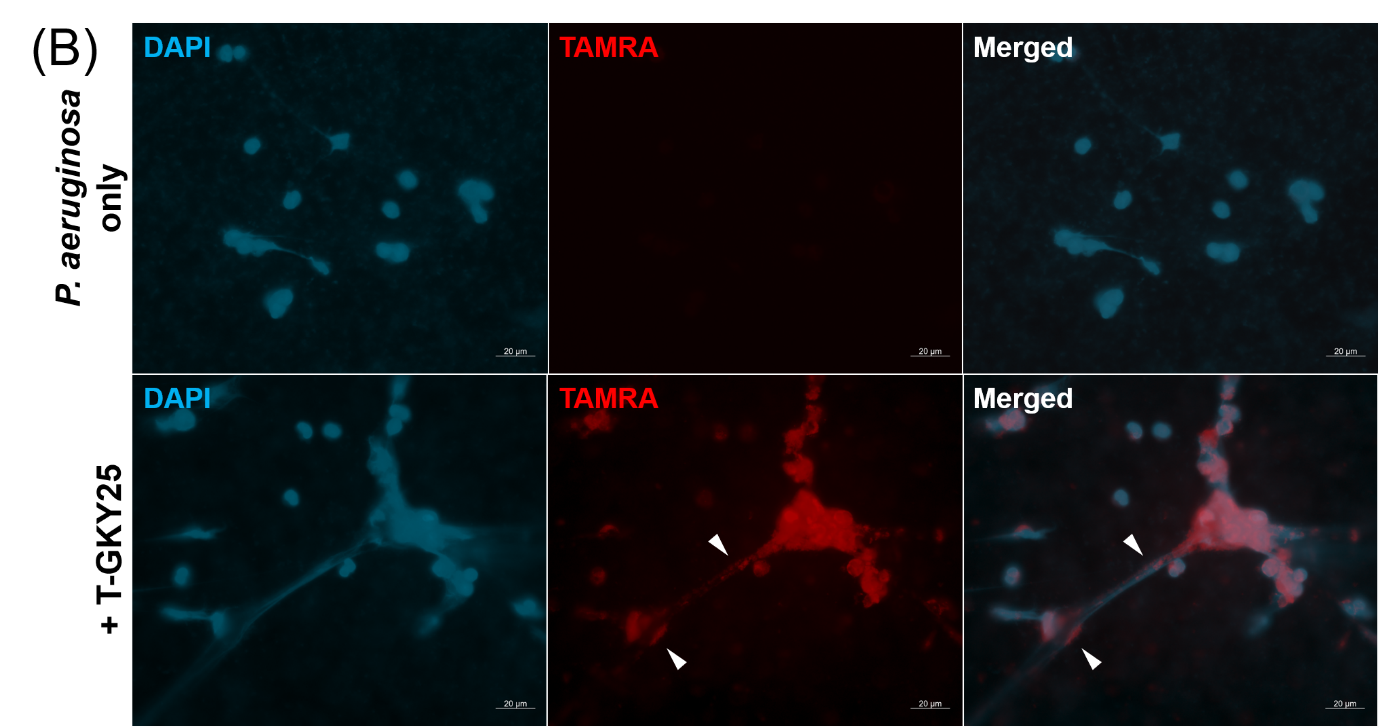


**Supp Figure 2. Thrombin C-terminal peptide binds NETs induced by different stimuli.** NETs were induced with **(A)** 5 µM of the calcium ionophore A23187 and **(B)** MOI 10 of the bacteria *P. aeruginosa* for 3 h at 37^o^C. After which, 5 µM of TAMRA-labelled GKY25 was added to both conditions for another 1 h at 37^o^C. White arrows mark represented colocalization between DNA/NETs and the peptide. Scale bar = 20 µm. Images are representative of 3 independent experiments.

Supplementary Figure 3


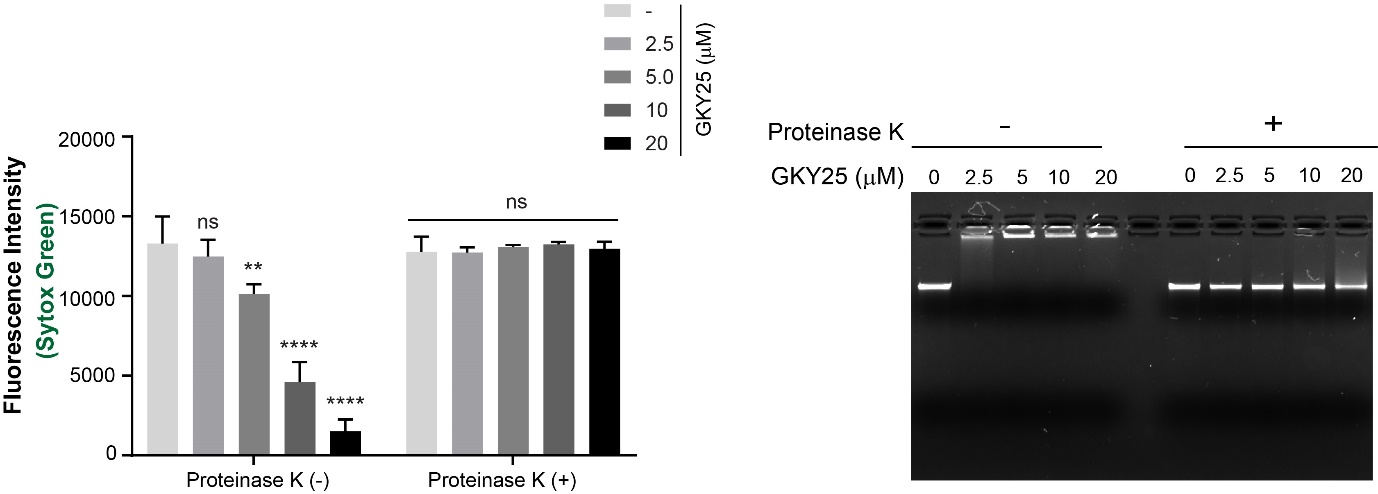


**Supp Figure 3. GKY25 DNA binding analysis** SYTOX green quantification (left) and agarose gel electrophoresis (right) of increasing concentrations of GKY25 (0, 2.5, 5, 10 or 20 µM) incubated with DNA (2 µg/ml) and then left untreated or treated with proteinase K (0.25 mg/ml) for 90 min at 37^o^C. Data represents the mean ± SD of n = 3 independent experiments. **p<0.01, ****p<0.001 by two-way ANOVA. This set-up was later utilized for the micrococcal nuclease protection assay as shown in Figure 5 of the main section.
